# Pulmonary sclerosing pneumocytoma: clinical features and prognosis

**DOI:** 10.1186/s12957-022-02603-4

**Published:** 2022-04-30

**Authors:** Quan Zheng, Jian Zhou, Guangchen Li, Shulei Man, Zhangyu Lin, Tengyong Wang, Boran Chen, Feng Lin

**Affiliations:** 1 Department of Thoracic Surgery, West China Hospital, Sichuan University, No. 37, Guoxue Alley, Chengdu, 610041 Sichuan China; 2 West China School of Medicine, Sichuan University, No. 37, Guoxue Alley, Chengdu, 610041 Sichuan China

**Keywords:** Pulmonary sclerosing pneumocytoma, Benign characteristic, Clinical features, Surgery

## Abstract

**Background:**

Pulmonary sclerosing pneumocytoma is a kind of rare benign pulmonary tumor with potential malignancy. The clinical features, risk factors for prognosis, and optimal treatment have not been identified yet. This study aimed to investigate the clinical features and prognosis of pulmonary sclerosing pneumocytoma.

**Methods:**

We retrospectively performed a review of pulmonary sclerosing pneumocytoma patients in West China Hospital from 2009 to 2019. The basic characteristics, treatment regimens, operation detail, postoperative variables, and follow-up time were recorded for each case. Differences in features between patients undergoing lobectomy and segmentectomy were compared. We also performed a case review and summarized reported clinical features in former studies.

**Results:**

Altogether 61 pulmonary sclerosing pneumocytoma patients were retrospectively reviewed. Fifty-six patients were female and 5 were male. The patients’ median age was 51 (23-73). Seven (11.48%) patients had smoking history. Twenty tumors were located in the right lung [upper lobe (*n* = 7), middle (*n* = 2), and lower (*n* = 11)] and 41 in the left [upper (*n* = 12) and lower (*n* = 29)]. The median tumor size was 2 (0.9-7) cm. Thirty-six (59.02%) patients underwent sublobectomy (segmentectomy or wedge resection) whereas 25 (40.98%) underwent lobectomy. All patients recovered uneventfully, and no perioperative mortality was identified. Sublobectomy showed a trend towards reduced chest tube duration and shorter postoperative hospital stays compared with lobectomy.

**Conclusions:**

The findings showed good prognosis of pulmonary sclerosing pneumocytoma and proved its benign characteristics. Sublobectomy showed advanced efficacy regarding chest tube duration and postoperative hospital stay compared with lobectomy.

## Background

Pulmonary sclerosing pneumocytoma (PSP), traditionally named pulmonary sclerosing hemangioma, is a kind of rare benign tumor with potential malignancy [1]. It was firstly described by Liebow et al. in 1956 [2]. Despite the implication by its name of a vascular neoplasm, sclerosing pneumocytoma was considered to be the tumor originated from pulmonary epithelium (type II pneumocyte) [3]. Therefore, some investigators called it as “pneumocytoma”. This kind of tumor was usually seen in the fifth-decade female [3], which was possibly attributed to the presence of progesterone receptors [1]. It was commonly presented as an asymptomatic solitary peripheral nodule [4] and an incidental lung mass on chest radiograms [5].

According to the WHO categorization of lung and pleural tumors (2018, ICD-11 for Mortality and Morbidity Statistics), pulmonary sclerosing pneumocytoma was categorized as benign, fibromatous neoplasms [6]. Nevertheless, there have been several reports on the possible malignant characteristics with lymph node metastasis [7–11] or local recurrence [12]. Although the prognosis of pulmonary sclerosing pneumocytoma seems not to be affected by these malignant potentials, there are other factors which may be related to the prognosis. The pulmonary sclerosing pneumocytoma have a large-scale range of size and could appear in different lobes of lung [13, 14]. However, whether these factors could affect the prognosis has not been discussed in a large cohort yet.

Furthermore, treatment for this kind of benign tumor remains controversial. Surgery was the main treatment for pulmonary sclerosing pneumocytoma [13, 14]. Sublobectomy, including mainly segmentectomy and wedge resection, tended to be preferred for peripheral small-sized tumor [7], while lobectomy could prevent the potential metastasis and recurrence which would worse long-term prognosis [15, 16]. However, which resection extent was optimal has not been answered well.

Herein, we retrospectively reviewed pulmonary sclerosing pneumocytoma patients admitted in our center from 2009 to 2019, aiming to investigate clinical features, risk factors, and treatment for patients with pulmonary sclerosing pneumocytoma.

## Methods

### Study cohort

**Fig. 1:**
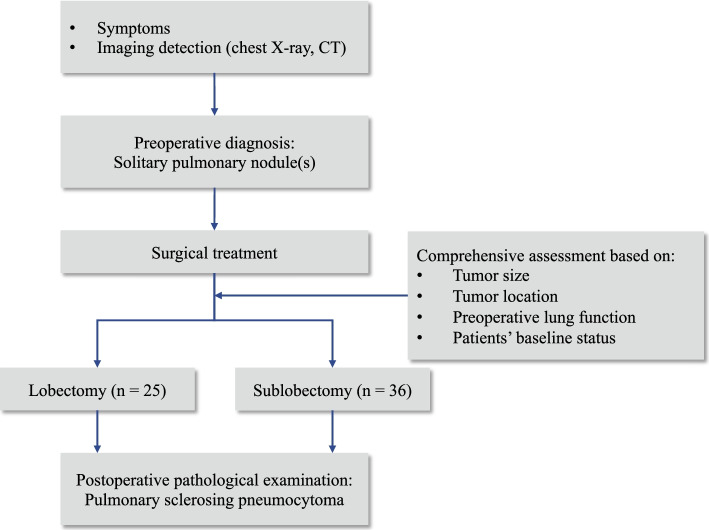
Flowchart showing diagnosis and management of patients in this study.

**Fig. 2:**
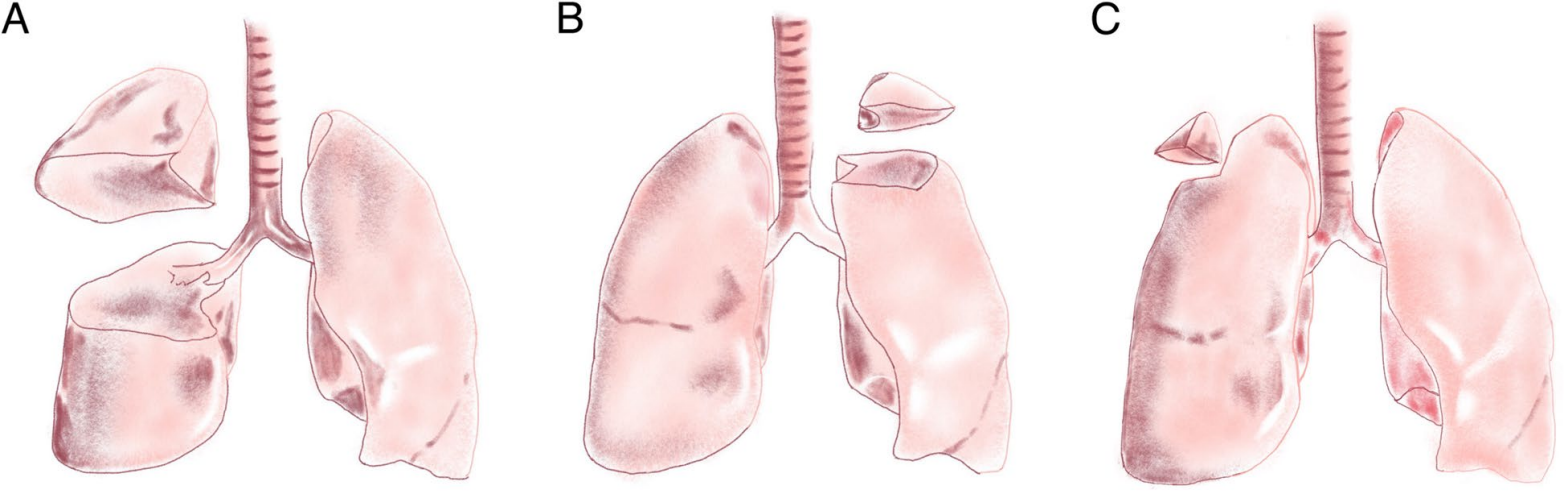
Representation image showing **A** lobectomy, sublobectomy: **B** segmentectomy and **C** wedge resection

We retrospectively performed a chart review of patients admitted in West China Hospital of Sichuan University from June 2009 to August 2019. Chest computed tomography (CT) was conducted among all patients, and those who were initially diagnosed with solitary pulmonary nodule and strongly asked for surgery would receive surgical treatment (Fig. 1). Resection extent of lobectomy or sublobectomy (Fig. 2) was decided based on the following criteria: (ii) tumor size; (ii) tumor location (peripheral versus central); (iii) preoperative lung function test; (iv) patients’ baseline characteristics like age and BMI. The decision was based on comprehensive consideration of the above criteria [17, 18]. All patients underwent intraoperative frozen section analysis, and being confirmedly diagnosed by postoperative pathological examination. We performed selective lobe-specific lymph node dissection and mediastinal lymph node dissection as the intraoperative frozen section showed non-malignancy of tumors. After surgery, we inspected and recorded chest drainage volume and air leak every day until chest tube removal. The removal criteria consisted of less than 300 mL drainage fluid/day, no bubbling was observed lasting 12 h, and adequate lung inflation in chest radiology. Postoperative complications were recorded during daily patient round. Patient was discharged the next day after chest tube removal as if no accidence existed.

The basic characteristics [demographic characteristics, smoking status, pulmonary function test outcomes, comorbidity (history of high blood pressure [HBP] and high glucose), tumor size, and location], treatment regimens, operation details (surgery duration and intraoperative blood loss), postoperative variables [length of postoperative hospital stay, chest tube duration and main postoperative complications (pulmonary infection, prolonged air leak [PAL], atelectasis, hoarseness, chylothorax, and bronchopleural fistula)] and follow-up time were collected. Tumor size was measured by the maximum diameter of tumor on preoperative CT scan. Chest tube duration was considered to be the time from chest tube placement to removal. PAL was considered as air leak lasting for more than 5 days. All indicators were defined according to the definition of the European Society of Thoracic Surgery and the Society of Thoracic Surgeons [19].

### Follow-up protocol

All patients received history inquiry, physical examination, chest, abdominal, and brain CT scans or magnetic resonance imaging (MRI) every 3 months for the first 1 year after surgery, every 6 months for 2 to 3 years, and every 12 months after 3 years. Besides, bone scan was performed every year until the last follow-up. Follow-up time was defined as the time from the day of surgery to the day of death or last follow-up.

### Literature review

We performed literature review on case series of PSP. We impose no limit on publication date. We summarized reported clinical features, including sex, age, tumor size, tumor location, extent of resection, and survival.

### Statistical analysis

All statistical analyses were performed using IBM SPSS (version 25.0, IBM Corp., Armonk, NY). Dichotomous variables were described as number of cases and incidence, while continuous variables as median (range) or mean ± standard difference. We then compared clinical features and early postoperative outcomes between two groups of sublobectomy and lobectomy. Furthermore, we compared incidence of complications in subgroups regarding to tumor location, sex, smoking history, and history of HBP. Dichotomous variables were analyzed using the Pearson chi-squared test, while continuous variables were compared using the Student *t* test. All tests were two-sided. *P* value less than 0.05 was considered statistically significant.

## Results

### Clinical features

From June 2009 to August 2019, 61 pulmonary sclerosing pneumocytoma patients were included in our study who underwent curative pulmonary resection in our institution. Intraoperatively, 56/61 patients were found to be pulmonary sclerosing pneumocytoma according to frozen section analysis, while 5 patients were diagnosed with benign lung neoplasm which could not be specified. The diagnoses of pulmonary sclerosing pneumocytoma for 61 patients were confirmed by postoperative pathological examination (Figs. 1 and 2). A total of 56 patients were female and 5 patients were male. The patients’ median age was 51(23-73) and the mean BMI was 22.69 (2.48) kg/m^2^. Altogether, 7 patients had a smoking history. Two patients had a history of hyperglycemia and 10 had a history of HBP. The median tumor size was 2 (0.9-7) cm. Altogether, 20 tumors were located in the right lung [upper lobe (*n* = 7), middle lobe (*n* = 2), and lower lobe (*n* = 11)] and 41 in the left [upper lobe (*n* = 12) and lower lobe (*n* = 29)]. The incidence of complications varied among different tumor location (*P* = 0.007). Table 1 summarized the clinical features of all included patients.

**Table 1:** Clinical characteristics of patients with pulmonary sclerosing pneumocytoma (*N* = 61)

Characteristics	All (*n* = 61)	Sublobectomy (*n* = 36)	Lobectomy (*n* = 25)	*P* value
Age, year				0.359
Mean ± SD	51.90 (12.40)	50.67 (12.13)	53.68 (13.06)	
Median (range)	51 (23-73)	51.5 (23-69)	55 (23-73)	
Sex, *n* (%)				1.000
Male	5 (8.20)	3 (8.33)	2 (8)	
Female	56 (91.80)	33 (91.67)	23 (92)	
BMI^a^, kg/m^2^	22.69 (2.48)	22.50 (2.19)	22.95 (2.92)	0.494
Smoking history, *n* (%)				0.430
Ever	7(11.48)	3 (8.33)	4 (16)	
Never	54(88.52)	33 (91.67)	21 (84)	
Comorbidity, *n* (%)				
Hypertension	10 (16.39)	5 (13.89)	5 (20)	0.727
Hyperglycemia	2 (3.28)	1 (2.78)	1 (4)	1.000
Pulmonary function				
FEV1^b^, L	2.33 (0.58)	2.27 (0.56)	2.41 (0.61)	0.186
FEV1/FVC^c^ (%)	77.41 (8.49)	76.52 (10.49)	78.67 (4.46)	0.278
Tumor size, cm				0.004^**^
Mean ± SD	2.87 (1.29)	2.48 (1.04)	3.44 (1.04)	
Median (range)	2 (0.9-7)	2.2 (0.9-5)	3 (2-7)	
Tumor location (lobe)				0.294
Left upper	12	7	5	
Left lower	29	17	12	
Right upper	7	2	5	
Right middle	2	2	0	
Right lower	11	8	3	

The data was presented as mean (SD) or median (range)

^**^P < 0.01. Continuous variables were compared using Student’s *t* test and categorical variables using chi-squared test

^a^ *BMI* body mass index

^b^ FEV1: the forced expiratory volume in 1 s

^c^ FEV1/FVC: the forced expiratory volume in 1 second (FEV1)/forced volume vital capacity (FVC) ratio

The median follow-up duration was 30 (2-95) months with a total of 3/61 patients lost follow-up. All patients recovered uneventfully, and no perioperative mortality was identified. All patients were free of local recurrence or distant metastasis during the follow-up period. The postoperative complications include pulmonary infection (1/61), prolonged air leak (2/61), and atelectasis (2/61).

### Pathological features

**Fig. 3:**
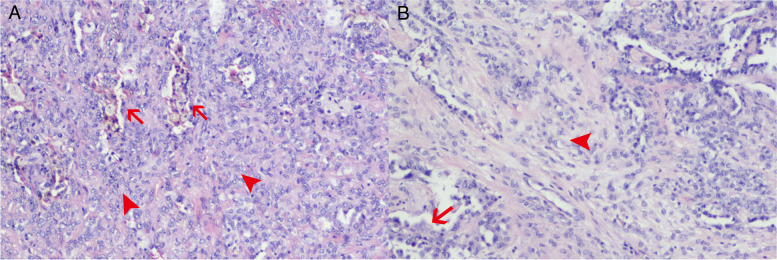
Histological section of pulmonary sclerosing pneumocytoma. **A** Hemorrhagic pattern (arrow) and solid pattern (arrowhead). **B** Papillary pattern (arrow) and sclerotic pattern (arrowhead). The arrow in **A** also pointed to superficial cubic cells

**Fig. 4:**
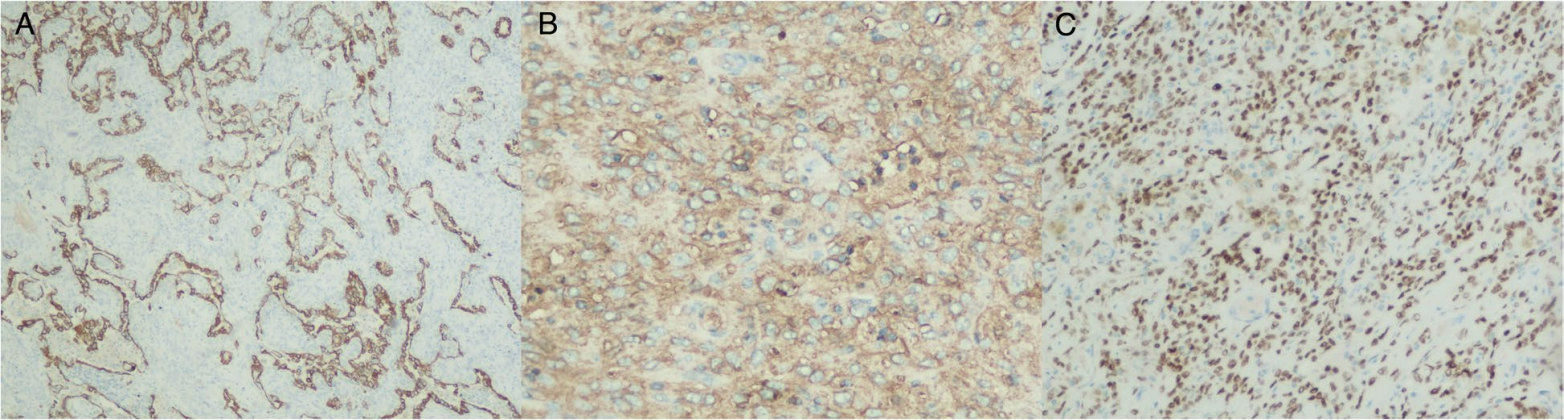
Immunohistochemical staining of pulmonary sclerosing hemangioma. **A** CK-7 positive in superficial cubic cells. **B** EMA immunolabeling positive in the interstitial round cells. **C** TTF-1 positive in both type of cells

In gross, the sclerosing pneumocytoma was well circumscribed, nonencapsulated, easily shelled out, solid and firm. The cut surface might be mottled or hemorrhagic. The histological morphology of sclerosing pneumocytoma is of large diversity, while there are mainly four different pathologic patterns (Fig. 3): papillary, solid, sclerotic, and hemorrhagic. Not all cases showed the whole four patterns, but mostly at least three patterns coexisted, and often one or two patterns were predominant. There were mainly two cell types under the light microscope (Fig. 3A). The first is interstitial round cells or polygonal cells, with relatively consistent morphology, rich and light staining of cytoplasm, indistinct cell borders, fine nuclear chromatin, and rare mitoses. This type of cells could protrude into the alveolar cavity to form a papillary pattern, or diffusely proliferate to form a solid pattern. The second type is superficial cubic cells with eosinophilic cytoplasm, small and hyperchromatic nuclei, covering the surface of the papilla and lining the irregular adenoid fissures and vascular luminal surfaces in solid areas. The commonly positive marker of immunohistochemical staining included EMA and TTF-1 in both types of cells, while CK7, Napsin in surface cells, and Vimentin, PR, ER in interstitial round cells (Fig. 4).

### Outcomes related to different surgical regimen

There are 36 (59.02%) patients underwent sublobectomy (segmentectomy or wedge resection), whereas 25 (40.98%) patients received lobectomy. The mean tumor size was 2.48 cm in sublobectomy group, while 3.44 cm in lobectomy group.

The difference of basic demographic characteristics between the sublobectomy and lobectomy group was well distributed regarding age, sex, BMI, preoperative comorbidities, smoking status, pulmonary function, and tumor location. The sublobectomy group had shorter surgery time than lobectomy group did (88.17 ± 26.49 minutes vs. 125.40 ± 41.64 min, *P* < 0.001). The less intraoperative blood loss was also noticed in the sublobectomy group compared with lobectomy group (47.92 vs. 79.20 mL, *P* = 0.006). All the surgical procedures were uneventful with no intraoperative severe bleeding or mortality. All the early postoperative outcomes of the 2 groups were showed in Table 2. Patients undergoing sublobectomy showed a trend towards reduced time to chest tube removal compared with lobectomy group [2(1-7) days, vs. 3(2-6) days, *P* < 0.001]. The median length of postoperative hospital stay in sublobectomy group was significantly shorter than that in the lobectomy group [4(2-10) days vs. 6(4-9) days, *P* = 0.003]. With respect to postoperative complications including pulmonary infection, prolonged air leak, and atelectasis, no significant difference was summarized between the sublobectomy and lobectomy groups. The median follow-up time was 28.5(7-95) months in sublobectomy group and 38.28(2-92) months in lobectomy group, no significant statistical difference was identified (*P* = 0.351).

**Table 2:** Clinical outcomes of patients with pulmonary sclerosing pneumocytoma (*N* = 61)

Characteristics	Sublobectomy (*n* = 36)	Lobectomy (*n* = 25)	*P* value
Surgery time, min	88.17 (26.49)	125.40 (41.64)	< 0.001^***^
Intraoperative blood loss, ml	47.92 (29.36)	79.20 (47.52)	0.006^**^
Chest tube duration, day	2 (1-7)	3 (2-6)	< 0.001^***^
Postoperative hospital stays, day	4 (2-10)	6 (4-9)	0.003^**^
Complications, *n* (%)	2 (5.56)	2 (8)	1.000
Pulmonary infection	0	1	0.410
Prolonged air leak	2	0	0.508
Atelectasis	1	1	1.000
Follow-up time, months	28.5 (7-95)	38.28 (2-92)	0.351

The data was presented as mean (SD) or median (range)

^**^P < 0.01

^***^P < 0.001. Continuous variables were compared using Student’s *t* test and categorical variables using chi-squared test

### Results of literature review

Altogether 27 articles reporting on case series of PSP were identified (Table 3). Age and sex were reported in all articles. The mean age in all reported patients was 49 (14.51) and ranged from 10 to 78 years old. The ratio for female was 81.7% (201/246). The mean (SD) of tumor size was 2.75 (1.95) cm among all reported patients and ranged from 0.5 to 12 cm.

**Table 3:** Results of literature review

Author	Years	Patients	Female	Age	Size, cm	Lobe location^a^					Extent of resection		Survival status		
						RUL	RML	RLL	LUL	LLL	Lobectomy	Sub-lobectomy	Lost to follow-up	Dead	Alive
K.W. Chan [20]	1982	14	14	50 (16.21)	2.33 (0.64)	0	2	5	4	1	9	1	6	1	7
A.S Fassina [21]	1986	6	3	59 (9.72)	1.83 (0.59)						4	2	1		5
N.P Ohori [22]	1991	14	14	45 (14.65)									3	3	8
A.C. Chan [23]	2000	1 6	15	52 (14.13)	2.39 (1.65)	1	5	4	0	6					
J.E. Nam [24]	2002	2	2	50 (16.26)	2.25 (1.06)	0	0	0	0	2					
M.C. Aubry [25]	2002	16	16	50 (12.65)	2.96 (2.35)						1	10	1	1	14
Y.C. Cheung [26]	2003	6	5	35 (14.58)							6	2			
K. Yamazaki [27]	2004	7	7	54 (14.30)	4.50 (3.22)										
S.D. Sak [28]	2007	26	16	53 (9.76)	2.08 (1.31)	5	2	3	2	14					
G. Sartori [29]	2007	11	10	47 (11.00)	3.09 (0.80)	1	2	0	4	4	5	6			11
S. Islam [30]	2009	6	3	56 (9.75)	1.92 (0.59)						1	1			
K.H. Lin [31]	2011	6	5	50 (11.29)	2.87 (1.55)	0	1	1	1	3					
Q.B. Wang [32]	2011	16	14	53 (11.66)		1	9	4	2	1					
Lee, E [33].	2013	26	23	44 (11.47)	2.47 (1.26)	2	6	6	2	8					
C.Y. Wu [34]	2016	14	14	53 (15.12)	2.26 (1.41)	2	2	2	3	5					
A. Lovrenski [35]	2019	6	5	51 (9.00)	1.75 (0.50)	0	0	1	2	3					6
J. Xu [36]	2019	22	12	54 (12.76)	2.59 (1.38)										
Q. Gao [37]	2020	32	23	41 (18.57)	4.77 (3.11)	4	1	9	6	12					
Overall		246	201	49 (14.51)	2.75 (1.95)	16	30	35	26	59	26	22	11	5	51
Median (range)				51(10-78)	2.20 (0.50-1.20)										

The data was presented as mean (SD) or median (range)

^a^ *LUL* left upper lobe, *LLL* left lower lobe, *RLL* right lower lobe, *RML*, right middle lobe, *RLL* right lower lobe

## Discussion

In most cases, pulmonary sclerosing pneumocytoma has a benign behavior. Shibata and colleagues [38] reported a patient with pulmonary sclerosing pneumocytoma that progressed into severe exertional dyspnea 47 years after detection of abnormal shadow through X-ray. The tumor measured 20 × 16 × 15 cm, weighed 2.3 kg, and occupied the whole left thoracic cavity. This case indicated that pulmonary sclerosing pneumocytoma was not self-limiting despite its benign nature. Some pulmonary sclerosing pneumocytoma cases have been reported with multiple lung involvement [39], lymph node metastasis [8, 11, 16], and distant metastasis [40]. Those elucidated the potential malignant nature of pulmonary sclerosing pneumocytoma. There were theories that lymph node metastasis of pulmonary sclerosing pneumocytoma was mediated through air space pattern [11]. Dantis and colleagues [11] reported that PET scan could help guide lymph node dissection, since the cases with SUV max uptake of more than 2.5 mostly had positive mediastinal lymph node metastasis. Wei and colleagues [4] reported a pulmonary sclerosing pneumocytoma case with local recurrence 10 years after initial wedge resection. This patient was subjected to a second wedge resection to completely remove the recurrent lesion. Iyoda and colleagues [12] also reported a pulmonary sclerosing pneumocytoma case with local recurrence 4 years after initial resection and therefore subjected to a second resection. However, these cases did not indicate a dismal prognosis. In our case series, all patients recovered uneventfully, and no perioperative mortality, postoperative recurrence, or metastasis was identified. In the review of prior studies, among 67 patients who reported survival status, 5 were reported of death. Overall, the biological behavior of pulmonary sclerosing pneumocytoma is obscure.

The molecular alterations in the sclerosing pneumocytoma were one of the study focuses and showed diagnostic value. *AKT1* mutation was the most commonly reported gene mutation and was speculated to be the genetic hallmark of sclerosing pneumocytoma [11, 41, 42]. *AKT1* mutation might induce cells proliferation and morphology changing, but would not induce progress to malignancy [41]. *Beta-catenin* was the secondly most common gene mutation in the sclerosing pneumocytoma [11, 41], which might also play a role in producing a benign tumor but not a malignant one. Mutations in other tumor-related genes were also identified in the sclerosing pneumocytoma, like *PTEN*, *BRAF V600E*, *BLM*, *KMT2D*, but with relatively smaller incidence than *AKT1* and *β-catenin* [11, 41, 43].

Pulmonary sclerosing pneumocytoma is more common in females and could occur in all ages. In our case series, female patients occupied 91% and patient age ranged from 23 to 73, while in the literature ratio of female was 81% and the age ranged from 10 to 78 years old. Previous studies have shown that the sclerosing pneumocytoma has no predilection for a particular lobe of the lung [14]. And there have not been any studies focusing on the prognostic effect of the tumor location. Our study showed that the incidence of complications varied among different tumor location (*P* = 0.007). Tumor located on right middle lobe showed a trend toward higher incidence of complications. We also evaluated other factors which may affect the early postoperative outcomes of the tumor, including sex, smoking history, and history of HBP; however, none of them showed relationship with prognosis.

There were few studies having a discussion on whether sublobectomy or lobectomy has better oncological outcomes for pulmonary sclerosing pneumocytoma [44]. We performed a comparison between the outcomes of the two surgical regimens in this case series. The distribution of age, preoperative morbidities, and lung function between patients with the two surgical regimens were well balanced. The sublobectomy (segmentectomy or wedge resection) showed a trend towards a better clinical outcome, with reduced time to chest tube removal and length of postoperative hospital stay compared with lobectomy for pulmonary sclerosing pneumocytoma patients. It meant that we could consider sublobectomy more for pulmonary sclerosing pneumocytoma, for it could not only remove the tumor completely, but also preserve lung function more.

This study had several limitations. First, the follow-up duration was limited. However, the long-term survival outcomes on pulmonary sclerosing pneumocytoma remained unclear, thus the optimal follow-up duration required was still uncertain. This exploratory study might provide the reference for follow-up in the further study. Considering its possible malignant characteristics, future studies could conduct a longer follow-up procedure (> 3 years) to observe outcomes (recurrence or metastasis) effectively. Second, limited sample size might restrict the sufficiency and efficiency of the conclusions. Researches with larger sample size was warranted. Third, as the potential selecting bias existed in comparison between two groups of patients with different surgical regimens (lobectomy or sublobectomy), the conclusion on outcomes different surgical regimen should be referenced cautiously. Larger clinical trials are expected to provide further analysis.

## Conclusion

Pulmonary sclerosing pneumocytoma showed benign behavior both in our case series and literature review. All patients in our case series recovered uneventfully without metastasis and recurrence. Both sublobectomy and lobectomy could achieve radical resection and present promising clinical outcomes. Sublobectomy showed a trend towards reduced chest tube duration and shorter postoperative hospital stay compared with lobectomy.

## Data Availability

The datasets generated during the current study are not publicly available but are available from the corresponding author on reasonable request.

## Abbreviations

PSP: Pulmonary sclerosing pneumocytoma
CT: Computed tomography
HBP: High blood pressure
PAL: Prolonged air leak
MRI: Magnetic resonance imaging
